# Association Between Betel Nut Chewing and Body Mass Index: A Cross-Sectional Study in Guam

**DOI:** 10.3390/ijerph22071006

**Published:** 2025-06-26

**Authors:** Michelle Nagata, Lindsey E. Merifield, Gabriela Cruz-Mattos, Allen Oamil, Xavier Heidelberg, Gertraud Maskarinec, Thaddeus A. Herzog, Yurii B. Shvetsov, Yvette C. Paulino, Brenda Y. Hernandez

**Affiliations:** 1 Population Sciences in the Pacific, University of Hawai’i Cancer Center, 701 Ilalo Street, Honolulu, HI 96813, USA; lmerifield@cc.hawaii.edu (L.E.M.); xkh@hawaii.edu (X.H.); therzog@cc.hawaii.edu (T.A.H.);; 2 School of Health, University of Guam, 303 University Drive, Mangilao, GU 96913, USA

**Keywords:** betel quid, areca nut, obesity, BMI, pacific islands

## Abstract

Areca nut/betel quid (AN/BQ), a stimulant consumed across the Asia and Pacific region, has been associated with metabolic risks including obesity. This study investigated the association between AN/BQ use and obesity in Guam. Participants included 120 men and women 18+ years old. Recruitment and interviews were conducted at a central dental clinic in Guam between July 2013 and October 2014. Multivariate general linear models were utilized to estimate the association of AN/BQ chewing with body mass index (BMI). Of the participants with a mean BMI of 30.4 (SD 6.9) kg/m^2^, 82.5% reported ever chewing AN/BQ. The mean adjusted BMI among AN/BQ chewers was 4.53 kg/m^2^ (95% CI 1.19, 7.87) higher than among non-chewers in the minimally adjusted model; 4.72 kg/m^2^ (95% CI 1.09, 8.35) higher with additional adjustment for annual household income, tobacco smoking, and alcohol use (n = 108); and non-significantly higher by 0.55 kg/m^2^ (95% CI −3.92, 5.02) after additional adjustment for ethnicity. Although AN/BQ chewing was not associated with BMI after considering ethnicity, our results do not exclude the possibility that AN/BQ chewing can be considered a risk factor for obesity.

## 1. Introduction

Guam is an island in Oceania, specifically Micronesia, located in the western North Pacific Ocean [1]. Guam has a diverse population composed of Chamorros (37.3%), Filipinos (26.3%), Whites (7.1%), Chuukese (7.0%), Koreans (2.2%), Chinese (1.6%), Palauans (1.6%), Japanese (1.5%), Pohnpeians (1.4%), other Pacific Islanders (2.0%), other Asians (2.0%), mixed ethnic groups (9.4%), and other ethnic groups (0.6%) [1,2].

Areca nut, the seed of the fruit of the *Areca catechu* palm tree, is ordinarily chewed, similarly to chewing tobacco. Areca nut is a stimulant consumed across the Asia and Pacific region and the fourth most common addictive substance globally. Often called “betel quid,” the areca nut is commonly combined with chewing tobacco and/or slaked lime (calcium hydroxide) and wrapped in a betel leaf (often from the *Piper betle*) to form a quid. Arecoline, the active substance found in the areca nut, acts as a nicotine receptor agonist [3]. Chewing tobacco and slaked lime are often added to areca nut/betel quid (AN/BQ) to increase the stimulant effect. Alcohol and other substances may also be added or consumed with AN/BQ use. Combined use may contribute to greater addiction instances than the areca nut alone [3,4].

Between 2011 and 2015, 11% of Guam’s population reported daily AN/BQ chewing [5]. Chamorros prefer chewing ripe areca nuts with betel leaf compared to other Micronesian ethnic groups. In contrast, Chuukese, Palauan, and Yapese prefer to chew unripe areca nuts in combination with slaked lime and tobacco products [6]. The addition of cardamom, ginger, and vodka to the betel quid has also been reported in Guam [5]. AN/BQ chewing has been identified as an independent risk factor for oral and esophageal cancers and may also play an etiologic role in liver and other cancers [7,8]. This increased risk may be due to the induction of oral submucous fibrosis, a premalignant state in the oral mucosa [9,10,11]. AN/BQ use is also associated with poorer oral cancer prognosis [11]. AN/BQ use is also linked to low birth weight and anemia during pregnancy, and a case study found that use during pregnancy may be linked to newborn neonatal abstinence syndrome, with betel nut found in the placenta [12,13].

This behavior may also be associated with overweight and obesity, with several studies reporting positive associations of AN/BQ use with obesity and metabolic syndrome [14,15,16] and others reporting slower increases in BMI with AN/BQ chewing [17]. BMI has been strongly correlated with adiposity and widely adopted as an indicator for obesity [18,19]. A transcriptomic study identified gene expression changes in a human monocyte-derived cell line (THP1) incubated with AN/BQ-derived arecoline relevant to obesity, type 2 diabetes, and metabolic syndrome [20]. The mechanism underlying the changes in BMI and metabolic rate still need to be elucidated. One potential theory is that certain alkaloids may inhibit the GABA receptors to increase appetite [15], though some research states that arecoline may act to suppress appetite [21]. Another mechanism through which AN/BQ use increases BMI is through increasing the risk of insulin resistance [15,22]. AN/BQ use has been associated with or found to worsen certain metabolic-associated diseases, including liver disease, asthma, diabetes, hypothyroidism, cardiovascular disease, hyperlipidemia, metabolic syndrome, and endocrine disorders [8,23,24].

Multiple studies have reported the prevalence and risks of AN/BQ use throughout the world. A narrative review found that multiple approaches are currently used for AN/BQ use cessation interventions: educational interventions in schools and for adults which raise awareness of the effects of AN/BQ or increase self-efficacy for quitting, pharmacological approaches employing antidepressants for people with AN/BQ use disorders and depressive disorders, and psychological interventions which are based on smoking cessation approaches [25]. The Betel Nut Intervention Trial (BENIT) study published in 2023 took place in Guam and Saipan, where the intervention group was provided a 22-day behavioral therapy program with an educational brochure, and the control group was provided only an educational brochure [26]. Compared to 9.1% of the control group, 38.6% of the intervention group stopped chewing [26]. The association between AN/BQ use and obesity has been understudied in Guam, a population with diverse ethnic groups, some of which consume areca nut in various forms. The purpose of this study was to analyze the relation of AN/BQ use with body mass index (BMI) after adjustment for demographic and lifestyle risk factors.

## 2. Materials and Methods

### 2.1. Study Design

The study population included participants from a prior study investigating the influence of AN/BQ chewing on the oral microbiome and oral premalignant lesions [27]. The prior study found that AN/BQ chewers demonstrated reduced richness and evenness and changes in the relative abundance of common bacteria, and microbiome alterations were most pronounced in current chewers [27]. Assessment of demographic, medical, and other lifestyle factors with AN/BQ use was limited, though current chewers had a higher BMI than past chewers and never-chewers [27]. Therefore, we designed the present study to further investigate the association between AN/BQ use and BMI within this study population. Briefly, the convenience sample included a total of 120 men and women recruited through study announcements and advertisements at a central dental clinic in Guam between July 2013 and October 2014. Individuals who were at least 18 years of age and able to understand English met the inclusion criteria. Recruitment and interviews were coordinated at the only periodontal clinic on the island at the time. At study enrollment, all participants completed a questionnaire detailing demographic, medical, and dietary history information. Questionnaire data were reviewed for completeness. The study was approved by the Institutional Review Boards at the University of Hawaii and the University of Guam. All participants provided written informed consent. A structured questionnaire was administered by research staff to collect information on demographics, medical history, smoking and alcohol use, and AN/BQ use, including the duration and frequency of consumption and the added use of tobacco use and alcohol consumption. Height and weight were measured and used to compute BMI. Given the many different ethnic groups represented in this study, four summary categories were created: Chamorros, other Pacific Islanders, Asians (11 Filipinos, 1 Japanese, 1 Chinese), and Whites.

### 2.2. Statistical Analysis

The frequency of having ever chewed AN/BQ was assessed across all study participants, as well as by demographic characteristics—sex, ethnicity (Chamorro, Other Pacific Islander, Asian, White), annual household income (< or ≥USD 50,000, education level (high school or less, college degree, graduate school), BMI category (<25, 25–<30, and ≥30 kg/m^2^), current tobacco smoking (yes/no) and alcohol use (yes/no)—using chi-square tests or Fisher’s exact tests (if cells had less than 5 individuals). Participants with missing sex or BMI were excluded from analyses. Dietary history information and frequency and duration of AN/BQ chewing were also excluded from analyses due to missing or incomplete data. Missing values of annual household income and frequency and duration of chewing were coded as a separate category. The association between BMI and ever/never AN/BQ chewing status was assessed using a series of general linear models, with Model 1 adjusted for participants’ age, sex, and education level; Model 2 additionally adjusted for household income, tobacco smoking, and alcohol use; and Model 3 also including ethnicity (four categories). Finally, we fit Model 4 after excluding Asian and White participants. Reference groups for each characteristic were defined as follows: AN/BQ non-chewers, male sex, education level of high school or less, annual household income of USD 50,000 or less, tobacco non-smokers, alcohol non-users, and Chamorro ethnic group. Beta-coefficients and 95% confidence intervals (95% CIs) were calculated. Covariate-adjusted means of BMI were calculated for AN/BQ chewers and non-chewers in Models 1 through 4. To examine the potential influence of two extreme BMI values on the model estimates, we conducted a sensitivity analysis with log-transformed BMI, which yielded nearly identical statistical significance and model fit statistics to those for the untransformed models. SAS Studio 3.81 (SAS Institute Inc., Cary, NC, USA) was used for statistical analyses.

## 3. Results

This study included 120 adult participants living in Guam sampled from a prior study investigating the influence of AN/BQ chewing on the oral microbiome and oral premalignant lesions for secondary data analysis [27]. The mean age was 41.0 (SD 15.1) years, and more than half of the participants (54.2%) were males. The majority (68%) had a college education (including 8.3% with a graduate degree) and 31.7% had a high school level education or less. Less than half of the participants (42.6%) had an annual household income of USD 50,000 or more. The mean BMI was 30.4 (SD 6.9) kg/m^2^ with nearly half of the participants classified as having obesity (46.7%) followed by overweight (35.8%) and healthy weight (17.5%). The majority of the participants consumed alcohol (55.8%), while only 29.2% of participants reported current tobacco smoking. As for AN/BQ chewing status, 82.5% of participants reported ever chewing (Table 1), and 62.2% of all participants were current chewers. Among AN/BQ chewers, 63.6% provided responses for the number of chews per day, frequency of chewing, and duration of chewing. Approximately equal proportions of chewers chewed fewer than 3 chews per day (34.9%), 3–10 chews per day (34.9%), and 10 or more chews per day (30.2%). The frequency of AN/BQ chewing included daily (60.3%), weekly (19.1%), and monthly (20.6%). More than half of the participants (54.0%) reported chewing AN/BQ for 20 years or more.

The proportion of AN/BQ chewers significantly differed by BMI (*p* = 0.03), ethnicity (*p* < 0.0001), and education level (*p* = 0.004; Table 1). A higher proportion of Chamorros (90.5%), other Pacific Islanders (92.9%), and Whites (80.0%) reported a history of AN/BQ chewing compared with no history of chewing, while a higher proportion of Asians (84.6%) reported no history of AN/BQ chewing compared with a positive history of AN/BQ chewing. The proportions of AN/BQ chewers among participants who reported an education level of high school or less (92.1%) and college (80.6%) were greater than the proportion of chewers among participants who reported a graduate school education (60.0%). AN/BQ chewers had a higher (*p* = 0.03) mean BMI of 31.0 (SD 6.9) kg/m^2^ than non-chewers, who had a mean BMI of 27.5 (SD 6.3) kg/m^2^.

The linear association between BMI and AN/BQ chewing status is presented in Table 2. In the first minimally adjusted model (n = 120), the mean BMI among AN/BQ chewers was 4.53 kg/m^2^ (95% CI 1.19, 7.87) higher than among non-chewers. The covariate-adjusted mean BMI was 31.27 kg/m^2^ (95% CI 29.41, 33.14) for chewers vs. 26.74 kg/m^2^ (95% CI 23.72, 29.76) for non-chewers. A significant negative association between age and BMI was detected, with a BMI decrease of 0.118 kg/m^2^ (95% CI −0.203, −0.033) per additional year of life.

In the second model with additional adjustments for annual household income, tobacco smoking, and alcohol use (n = 108), the difference in mean BMI between AN/BQ chewers and non-chewers increased to 4.72 kg/m^2^ (95% CI 1.09, 8.35). The covariate-adjusted mean BMI was 30.85 kg/m^2^ (95% CI 28.76, 32.94) for chewers and 26.13 kg/m^2^ (95% CI 22.77, 29.49) for non-chewers. Age remained significantly associated with BMI, with a BMI decrease of 0.139 kg/m^2^ (95% CI −0.238, −0.040) per additional year.

Adding ethnicity to the third model (n = 108) attenuated the association between AN/BQ chewing status and BMI. AN/BQ chewers showed a non-significant increase in adjusted mean BMI of 0.550 kg/m^2^ (95% CI −3.92, 5.02) compared with non-chewers, with respective covariate-adjusted means of 29.0 kg/m^2^ (95% CI 26.26, 31.72) for chewers vs. 28.44 kg/m^2^ (95% CI 24.59, 32.29) for non-chewers. However, age remained significantly associated with BMI with a decrease of 0.127 kg/m^2^ (95% CI −0.227, −0.028) per 1-year increase. Compared with Chamorros, Asians were the only ethnic group with a significantly different mean BMI, which was 7.12 kg/m^2^ (95% CI −12.44, −1.80) lower for Asians compared with Chamorros.

To further examine the attenuated association between BMI and AN/BQ chewing observed in Model 3, the fully adjusted Model 4 restricted to Chamorros and other Pacific Islanders (n = 90) showed that the mean BMI was non-significantly lower by 0.256 kg/m^2^ (95% CI −5.66, 5.14) among AN/BQ chewers compared with non-chewers. The covariate-adjusted mean BMI was 30.68 kg/m^2^ (95% CI 28.12, 33.24) for chewers and 30.93 kg/m^2^ (95% CI 25.35, 36.52) for non-chewers. Age remained significantly associated with BMI, with a lower BMI of 0.619 (95% CI −0.281, −0.058) kg/m^2^ per additional year. No significant associations of BMI with sex, education level, annual household income, current tobacco smoking, or alcohol use were observed in any of the models. In summary, Table 2 reveals a significant association between AN/BQ chewing and BMI in Models 1 and 2. However, the inclusion of ethnicity into Models 3 and 4 attenuated the association of AN/BQ chewing with BMI to statistical non-significance.

## 4. Discussion

In this small study population with a wide range of ethnic groups from Guam, a significant correlation between AN/BQ chewing and BMI was found in a minimally adjusted model. The adjusted mean BMI was nearly 5 kg/m^2^ higher among participants who reported AN/BQ chewing than those who did not. However, after adjustment for ethnicity and restriction of the analysis to Pacific Islanders only, the association disappeared. This observation suggests that the higher BMI previously observed for chewers was due to the small subgroup of Asians with low BMI and a low frequency of AN/BQ chewing. Therefore, the current study sample does not show a correlation of BMI with AN/BQ use in Guam among Pacific Islanders.

This contrasts with studies from several countries including Taiwan, Myanmar, and Malaysia that found positive associations of AN/BQ use with obesity and metabolic syndrome, which includes body weight as one component [14,15,16]. In Myanmar, the risk of metabolic syndrome was positively associated with frequency and duration of chewing, doubling for men who chewed ≥ 10 pieces of AN/BQ per day (OR 2.49, 95% CI 1.36, 4.57) and for ≥10 years (OR 2.15, 95% CI 1.21, 3.84) compared with non-chewers [14]. In a Taiwan study that evaluated metabolic syndrome components at baseline and follow-up examination after a median of 4 years, having a history of AN/BQ chewing was significantly associated with baseline abdominal obesity (OR 1.55, 95% CI 1.46, 1.64) and with baseline metabolic syndrome after adjusting for smoking and alcohol use (OR 1.90, 95% CI 1.47, 2.46) [15]. Furthermore, a long duration of AN/BQ chewing was associated with baseline abdominal obesity (OR 1.01, 95% CI 1.00, 1.01) and low baseline metabolic syndrome (OR 1.01, 95% CI 1.00, 1.01) [15]. Another study in Taiwan reported that AN/BQ chewing was an independent risk factor for general obesity (OR 1.43, 95% CI 1.07, 1.91) and central obesity (OR 2.27, 95% CI 1.53, 3.37) [16]. Waist circumference (*p* = 0.02) and waist-to-height ratio (*p* = 0.02) were also higher in current chewers than in former chewers [16]. In Malaysia, AN/BQ chewers had higher BMI but slower increases in BMI from 1990 to 1996 compared with non-chewers, though these differences were not statistically significant [17,28]. These discrepancies in the relationship between AN/BQ use and obesity might be addressed by implementing a prospective design and considering ethnicity when evaluating this association in future studies.

Betel nut use has also been linked to a high incidence of cardiovascular disease [23] and found to aggravate diabetes and cardiovascular disease [23,24,28], but the current study did not explore associations with other metabolic conditions. Looking into additional health conditions associated with betel nut use in the future may reveal additional health concerns. Previous research in Taiwan found an increased prevalence of obesity with an increased duration of betel nut use [15]. Further analysis into the duration and intensity of chewing may reveal associations with BMI. However, given the sample limitations on duration and intensity, further analysis with this dataset was not appropriate. Physiological mechanisms for metabolism disruption and betel nut use are poorly understood. However, some potential mechanisms have been explored [29]. A study using mouse models found arecoline, the active ingredient in betel nut, promoted lipid metabolism in addition to affecting inflammation, oxidative liver stress, and intestinal injury microbiome implications [30].

The lack of association between annual household income and AN/BQ use indicates that use in Guam is not predominant in a certain economic class or marital status. This is not surprising considering the longstanding and often cultural significance of betel nut [24]. However, education level was a predictor of AN/BQ chewing, which raises the possibility that individuals with more schooling may have a better understanding of the risk associated with this behavior [31]. Obesity is a complex health concern with multiple risk factors, as noted in a recent study on obesity in Guam [32]. Measurements for fat mass, visceral fat content, and body fat distribution were not available in this study population and are recommended to further evaluate the relationship between adiposity and AN/BQ chewing on Guam. Lifestyle factors not analyzed in this study, such as diet, physical activity, and other health conditions may play a greater role than betel nut use in obesity prevalence.

Additional limitations to this study include the small sample size with several ethnic groups that were represented by only a few individuals. Ethnic groups were pooled into four summary categories, resulting in only four ethnic groups representing our final study population. This limitation may have reduced statistical power and generated inaccurate population estimates. Our study is also limited by the use of a standardized BMI for different ethnic groups. Given that the participants were recruited from dental clinics to study the oral microbiome of AN/BQ chewers [27], the small proportion of non-chewers and normal-weight participants led to selection bias. Therefore, the study population does not represent the general population and the results may not apply to other settings. These findings also demonstrate the problems in a secondary analysis of data collected for a different purpose, in particular in a convenience sample that may not reflect current AN/BQ usage and other lifestyle factors. However, strengths of this study are the availability of data, which was obtained by request to the Pacific Island Partnership for Cancer Health Equity, for an understudied population in the Pacific addressing a serious health behavior [33].

## 5. Conclusions

In conclusion, in this sample of Guam residents, AN/BQ chewing was associated with BMI using the entire sample, but controlling for ethnic background eliminated the association. Our findings highlight the importance of considering ethnicity when evaluating this association and support previous studies suggesting that AN/BQ chewing may be a risk factor for obesity.

## Figures and Tables

**Table 1:** Demographic and lifestyle characteristics of Guam study participants by betel nut chewing status (July 2013 to October 2014).

Characteristic	Category	Betel Nut Chewers (%) ^a^	Non-Chewers (%) ^a^	P ^b^
Number		99 (82.5%)	21 (17.5%)	–
Mean age, years (SD)		42.0 (14.7)	36.0 (16.5)	0.13
BMI, kg/m^2^ (SD)		31.0 (6.9)	27.5 (6.3)	0.03
BMI, kg/m^2^	<25	12 (57.1%)	9 (42.9%)	0.003
	25 to <30	40 (93.0%)	3 (7.0%)	
	≥30	47 (83.9%)	9 (16.1%)	
Ethnicity	Chamorro	67 (90.5%)	7 (9.5%)	<0.0001
	Other Pacific Islander	26 (92.9%)	2 (7.1%)	
	Asian	2 (15.4%)	11 (84.6%)	
	White	4 (80.0%)	1 (20.0%)	
Sex	Male	55 (84.6%)	10 (15.4%)	0.51
	Female	44 (80%)	11 (20%)	
Annual household income	<USD 50,000	52 (83.9%)	10 (16.1%)	0.64
	≥USD 50,000	37 (80.4%)	9 (19.6%)	
Education level	High school or less	35 (92.1%)	3 (7.9%)	0.004
	College	58 (80.6%)	14 (19.4%)	
	Graduate school	6 (60.0%)	4 (40.0%)	
Current tobacco smoking	No	69 (81.2%)	16 (18.8%)	0.55
	Yes	30 (85.7%)	5 (14.3%)	
Alcohol use	No	42 (79.2%)	11 (20.8%)	0.40
	Yes	57 (85.1%)	10 (14.9%)	

^a^ Counts and percentages exclude cases with unknown characteristics. Percentages correspond to betel nut chewing status. ^b^ *p*-values were obtained by Student’s *t*-test, Chi-square test, and Fisher’s exact test (when cells were smaller than n = 6).

**Table 2:** Association of ever/never betel nut chewing behavior and other factors with BMI among Guam study participants (July 2013 to October 2014).

Characteristic ^a^	Model 1	Model 2	Model 3	Model 4
	Beta-Coefficient (95% CI) ^b^			
Betel nut chewing vs. non-chewing	4.53 (1.19, 7.87)	4.72 (1.09, 8.35)	0.55 (−3.92, 5.02)	−0.26 (−5.66, 5.14)
Age, years	−0.12 (−0.20, −0.03)	−0.14 (−0.24, −0.04)	−0.13 (−0.23, −0.03)	−0.62 (−0.28, −0.06)
Female vs. male	1.62 (−0.81, 4.06)	2.40 (−0.50, 5.30)	1.99 (−0.83, 4.81)	2.51 (−0.59, 5.61)
Graduate school vs. high school or less	0.61 (−4.45, 5.67)	−0.26 (−5.69, 5.17)	0.98 (−4.38, 6.34)	−1.81 (−7.98, 4.36)
College vs. high school or less	0.53 (−2.16, 3.23)	−0.37 (−3.45, 2.72)	0.362 (−2.71, 3.43)	0.98 (−2.20, 4.16)
Income ≥ USD 50,000 vs. <USD 50,000	-	−0.11 (−3.00, 2.78)	−0.06 (−2.88, 2.76)	−0.47 (−3.59, 2.65)
Current smoking vs. non-smoking	-	−2.49 (−5.71, 0.74)	−1.93 (−5.12, 1.26)	−1.65 (−5.13, 1.83)
Alcohol use vs. not	-	1.23 (−1.88, 4.35)	1.53 (−1.57, 4.6)	0.56 (−2.83, 3.95)
Other Pacific Islander vs. Chamorro	-	-	1.95 (−1.65, 5.54)	0.97 (−2.81, 4.74)
Asian vs. Chamorro	-	-	−7.12 (−12.4, −1.80)	-
White vs. Chamorro	-	-	−2.35 (−8.51, 3.81)	-
	Covariate-adjusted mean (95% CI)			
Betel nut chewing	31.3 (29.4, 33.1)	30.9 (28.8, 32.94)	29.0 (26.3, 31.7)	30.7 (28.1, 33.2)
Non-betel nut chewing	26.7 (23.7, 29.8)	26.1 (22.8, 29.5)	28.4 (24.6, 32.3)	30.9 (25.4, 36.5)

^a^ Reference groups for each characteristic were AN/BQ non-chewers, male sex, education level of high school or less, annual household income of USD 50,000 or less, tobacco non-smokers, alcohol non-users, and Chamorro ethnic group. ^b^ Obtained by general linear regression with BMI as the dependent variable and betel nut chewing as the independent variable adjusted for covariates as follows: Model 1: age, sex, education (n = 120). Model 2: age, sex, education, income, smoking, alcohol (n = 108, income has 12 missing). Model 3: age, sex, education, income, smoking, alcohol, ethnicity (n = 108). Model 4: age, sex, education, income, smoking, alcohol, other Pacific Islander vs. Chamorro only (n = 90).

## Data Availability

The data presented in this study are available on request from the corresponding author. The data are not publicly available due to privacy restrictions.
